# Reversible senescence of human colon cancer cells after blockage of mitosis/cytokinesis caused by the CNF1 cyclomodulin from *Escherichia coli*

**DOI:** 10.1038/s41598-018-36036-5

**Published:** 2018-12-12

**Authors:** Zhen Zhang, Kyaw Min Aung, Bernt Eric Uhlin, Sun Nyunt Wai

**Affiliations:** 1 Department of Molecular Biology and The Laboratory for Molecular Infection Medicine Sweden (MIMS), Umeå University, SE-90187 Umeå, Sweden; 2 Present Address: Department of Food Hygiene and Environmental Health, Faculty of Veterinary Medicine, University of Helsinki, FIN-00014 Helsinki, Finland

## Abstract

Cytotoxic necrotizing factor 1 (CNF1), a protein toxin produced by extraintestinal pathogenic *Escherichia coli*, activates the Rho-family small GTPases in eukaryotic cell, thereby perturbing multiple cellular functions. Increasing epidemiological evidence suggests a link between CNF1 and human inflammatory bowel disease and colorectal cancer. At the cellular level, CNF1 has been hypothesized to reprogram cell fate towards survival due to the role in perturbing cell cycle and apoptosis. However, it remains undetermined how cells survive from CNF1 intoxication. In this work, we show that CNF1 treatment blocks mitosis/cytokinesis, elicits endoreplication and polyploidisation in cultured human colon cancer cells, and drives them into reversible senescence, which provides a survival route for cells via depolyploidisation. Senescence in CNF1-treated cells is demonstrated with upregulation of several senescence markers including senescence-associated β-galactosidase activity, p53, p21 and p16, and concomitant inhibition of the retinoblastoma protein phosphorylation. Importantly, progeny derived from CNF1 treatment exhibit genomic instability exemplified by increased aneuploidy and become more resistant to CNF1, but not to 5-fluorouracil and oxaliplatin, the two agents commonly used in chemotherapeutic treatment for colorectal cancer. These observations display survival features of the cell after CNF1 treatment that may have implications for the potential role of CNF1 in carcinogenesis.

## Introduction

Many pathogenic bacteria produce virulence factors to target host GTP-binding proteins, especially Rho-family small GTPases^1,2^. Due to the conversion between active GTP-bound and inactive GDP-bound conformation, Rho GTPases serve as molecular switches that regulate actin cytoskeleton and other cellular functions including cell cycle, polarity, migration, cytokinesis, proliferation, apoptosis and survival^3^. Through either activating or inhibiting the function of Rho GTPases, pathogenic bacteria disrupt host epithelial barrier and facilitate efficient colonization^4–6^.

Cytotoxic necrotizing factor-1 (CNF1), the first bacterial toxin found to activate host cell Rho family GTPases including RhoA, Rac1, and Cdc42 by inducing glutamine deamidation, is frequently associated to extraintestinal pathogenic *Escherichia coli* (ExPEC) strains of phylogenetic groups B2 and D that cause urinary tract infection (UTI) and other extraintestinal diseases such as meningitis and septicemia^7–12^. CNF1 is reported to contribute *E. coli* invasion of brain endothelial cells and penetration into the brain in an experimental rat meningitis model^13^. The role of CNF1 in the pathogenesis of UTI remains obscure. *In vivo* studies using a mouse infection model suggest that a CNF1-producing *E. coli* strain causes persistent colonization in the lower urinary tract with extensive inflammation compared to infection with a *cnf1* isogenic mutant^14,15^. However, studies using similar methods showed that the contribution of CNF1 in UTI caused by *E. coli* is not significant^16,17^.

At the cellular level, CNF1 was shown to be internalised after binding to the surface of epithelial cells, by receptor-mediated endocytosis and subsequently transferred to an endosomal compartment of the target cells^18–20^. CNF1 induces reorganisation of actin cytoskeleton and assembly of actin stress fibers, lamellipodia and filopodia^10,21^. Multinucleation and cell shape enlargement are common morphologic changes observed in various cell lines after prolonged treatment with CNF1, probably as a result from CNF1-induced mitosis/cytokinesis failure^10,21,22^. CNF1 is classified as cyclomodulin due to its role in perturbation of host cell cycle^23–25^. It was shown that CNF1 prevents the CDK1-cyclin B1–dependent cell cycle progression and arrests cells at G2/M phase^26,27^. Early studies also showed that CNF1 stimulates DNA synthesis and promotes the transition of quiescent cells into proliferation^21,28^. Several studies pointed out a link of cyclomodulin-producing *E. coli* to human inflammatory bowel disease and colorectal cancer^29–32^. A significant higher rate of CNF1-producing *E. coli* strains were identified in gut mucosa of patients with colon cancer (39.5%) than in those of patients with diverticulosis (12.9%)^31^, suggesting that CNF1 might participate into human colon carcinogenesis during chronic infection. Interestingly, a recent study described that CNF1 plays a role in prostatic carcinogenesis and prostate cancer (PCa) progression by activating a Cdc42–PAK1 signal axis and up-regulating the expression of MMP-9^33^. Earlier studies demonstrated multiple roles of CNF1 in cell signaling, such as counteracting apoptosis, and inducing production of pro-inflammatory cytokines, COX2 expression, and NF-kB activation^34–37^. Based on these findings, CNF1 is proposed to reprogram the cell fate towards survival^22,23,25,38^. The process of cell survival from CNF1 intoxication^22^, however, has not been thoroughly investigated. What survival strategy is utilized by cells to counteract CNF1 intoxication and facilitate proliferation remains unclear.

In the present study, we show that CNF1 blocks cell mitosis/cytokinesis in human colon cancer cell line, triggers endoreplication and destines cells to multinucleation, polyploidy and reversible senescent arrest. These events ultimately are followed by depolyploidisation-associated survival to generate genomically unstable progeny.

## Results

### Human colon cancer cells undergo endoreplication and polyploidisation in response to CNF1 treatment

**Figure 1:**
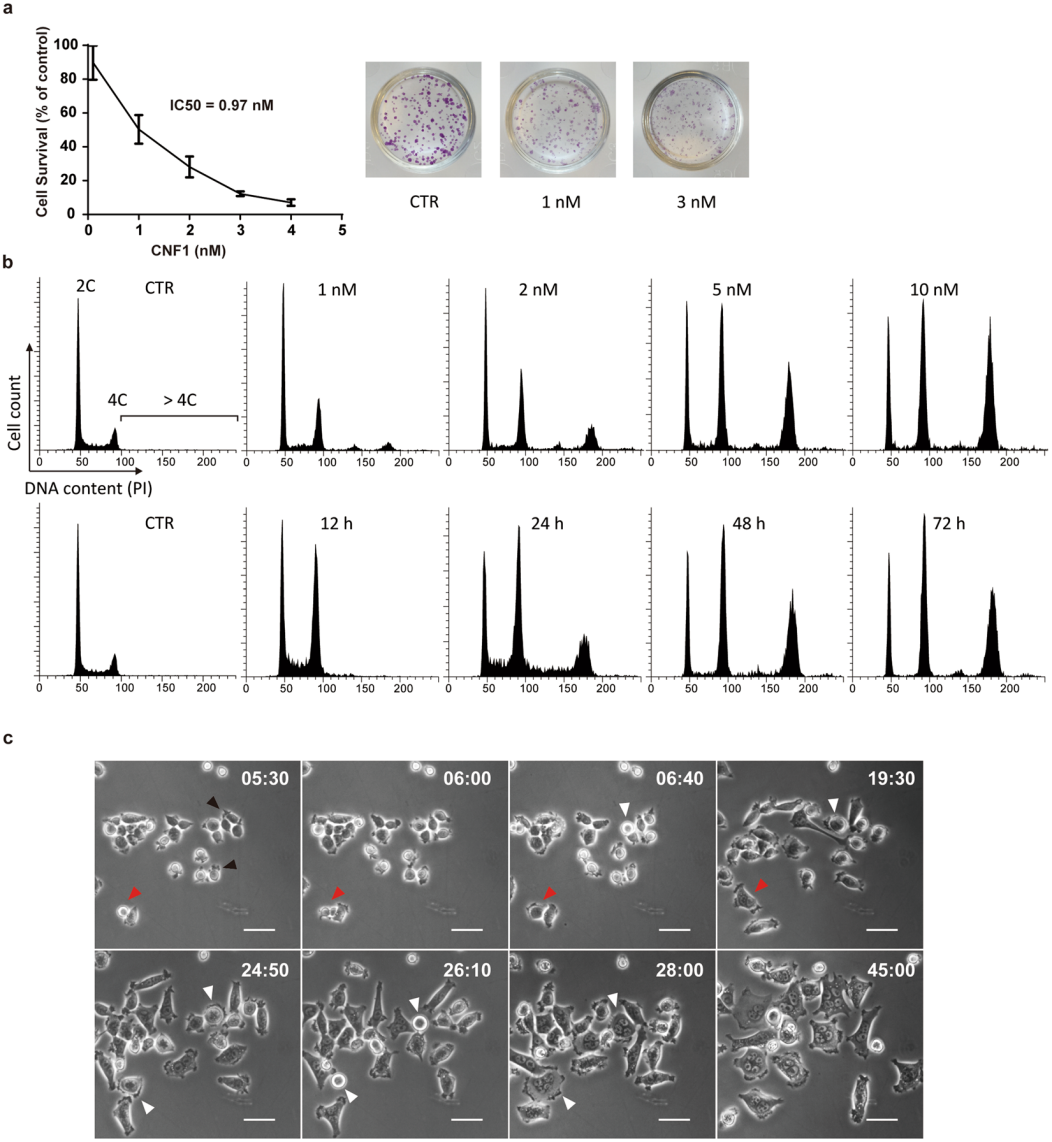
Endoreplication and polyploidisation in CNF1-treated HCT-116 cells. (**a**) Clonogenic assay for the determination of IC50 of CNF1 in HCT-116. Data are mean ± SD of three different experiments. The right panel shows representative pictures of colony formation in untreated cells (control, CTR) and cells treated with different concentrations of CNF1. (**b**) DNA content analysis of HCT-116 cells treated with increasing concentrations of CNF1 for 72 h (upper panel) or 5 nM CNF1 at different time points during 72 h (lower panel). (**c**) Representative time-lapse images of CNF1-induced endoreplication in HCT-116. Time is indicated in hours and minutes (h:min). Black arrowheads indicate a filopodium-like structure at the cell periphery; Red arrowheads indicate a cytokinesis failure; White arrowheads indicate continuous endoreplication and multinucleation. Bars, 50 μm.

We first evaluated the effect of CNF1 on proliferation of human colon cancer cells (HCT-116) using a clonogenic assay. When cells were plated at low density and treated with different concentrations of CNF1 for 10 days, the colony formation of HCT-116 decreased with increasing CNF1 concentration. The half maximal inhibitory concentration (IC50) of CNF1 was 0.97 nM in HCT-116 (Fig. 1a). To test the effect of CNF1 on cell cycle, we measured DNA content of cells after 72 h treatment with different CNF1 concentrations from 1 nM to 10 nM. In comparison to untreated cells, the proportion of polyploid cells (DNA content >4 C) significantly increased after exposure to increasing concentrations of CNF1 (Fig. 1b), suggesting that CNF1 induces cell polyploidisation in HCT-116. Diploid cells (DNA content 4 C) also increased largely whereas haploid cells (DNA content 2 C) only decreased slightly in treatment with high CNF1 concentration. We decided to treat HCT-116 cell with 5 nM CNF1 in this study, with special emphasis on CNF1-induced polyploid cells. We then measured the time course of cell polyploidisation in HCT-116 cells after treatment with 5 nM CNF1. The proportion of polyploid cells (DNA content >4 C) increased considerably from 12 h to 48 h after CNF1 treatment and maintained at 72 h. Diploid cells (DNA content 4 C) also increased whereas haploid cells only decreased slightly during treatment. The results indicate that CNF1 induces cell polyploidisation in dosage- and time-dependent manner.

It is known that CNF1 causes cell multinucleation and endomitosis in various cell lines (Boquet 2001). Therefore, it is conceivable that multinucleated polyploidy is a result of cells undergoing the variant cell cycle, endoreplication. To confirm that, we tracked the time course of morphologic changes of the HCT-116 cells after CNF1 treatment using time-lapse microscopy. We observed that the lethal dose (5 nM) of CNF1 blocked cell division in a quick and efficient manner (Fig. 1c and Supplementary Movie S1). Most of HCT-116 cells first exhibited a small, condensed and rounding shape with local and irregular filopodium-like structure at the cell periphery (Fig. 1c, 5 h 30 min [05:30], black arrowheads) and then turned into big and flattened cell shape with multinuclei (45:00). The regular cell division was halted mostly at the stage of mitotic rounding and then switched endoreplication (06:40 and 19:30, white arrowheads). Meanwhile, the failure of separation of two daughter cells at the stage of cytokinesis followed by endoreplication was also observed (from 05:30 to 19:30, red arrowheads). Endoreplicated cells underwent further endoreplication, resulting in increased nucleus number and cell shape (from 24:50 to 28:00, white arrowheads). By contrast, untreated HCT-116 cells had normal cell proliferation, did not show any significant morphologic changes (Supplementary Fig. S1 and Movie S2).

Combining DNA content analysis and real-time cell morphology evaluation suggests that CNF1 switches cells from normal cell cycle to endoreplication, resulting in multinucleated polyploidisation.

### CNF1-treated cells survive through depolyploidisation

**Figure 2:**
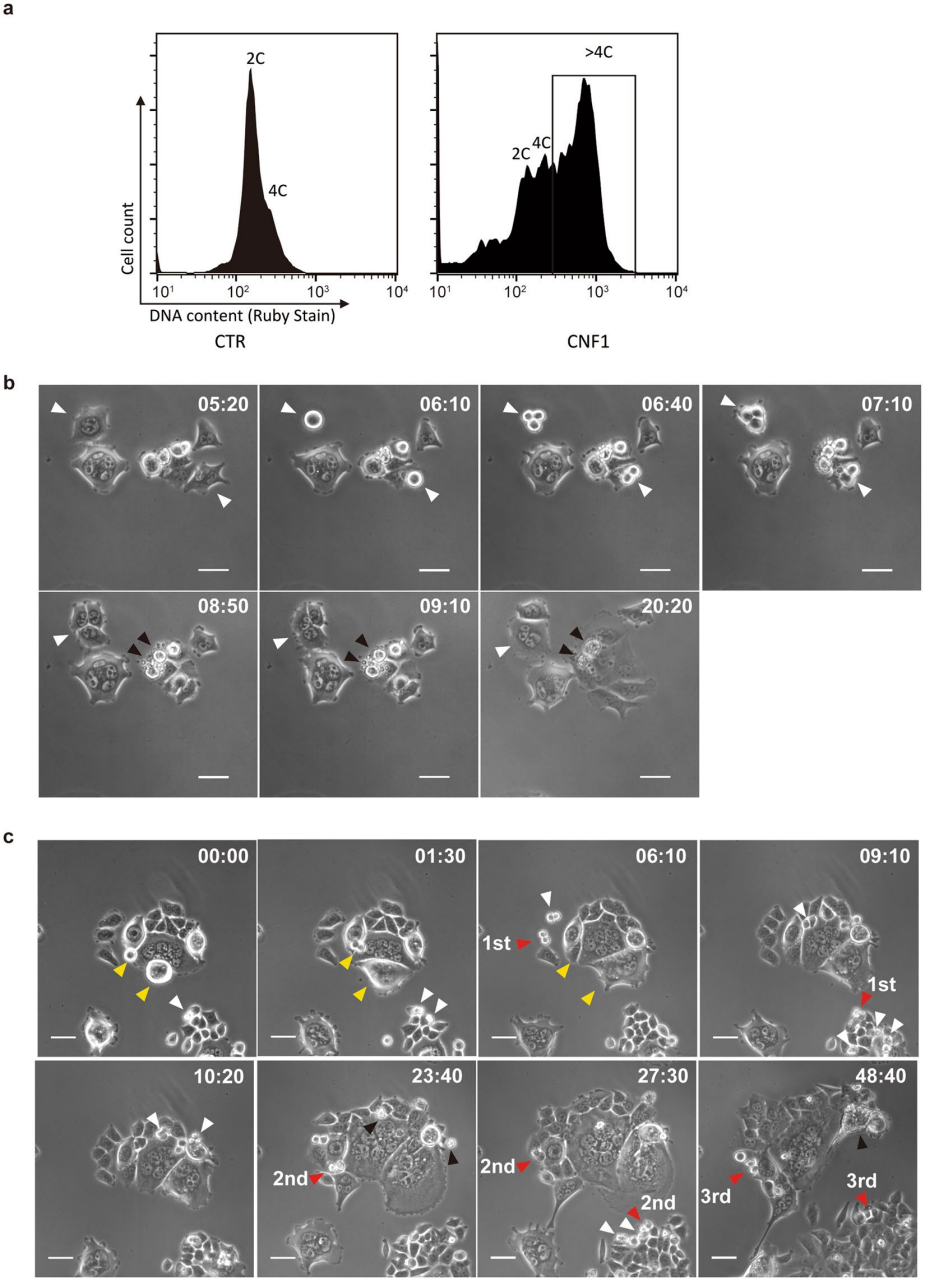
Survival of polyploid HCT-116 cells through depolyploidisation. (**a**) FACS sorting of polyploid HCT-116 cells (DNA content >4 C) after treatment with 5 nM CNF1. (**b**) Representative time-lapse images of depolyploidisation of polyploid HCT-116 cells in the first 7 days after CNF1 treatment. Time is indicated in hours and minutes (h:min). White arrowheads indicate successful mitotic re-entry and cell division; Black arrowheads indicate failed mitotic re-entry and cell death. Bars, 50 μm. (**c**) Representative time-lapse images of depolyploidisation of polyploid HCT-116 cells in second 7 days after CNF1 treatment. Time is indicated in hours and minutes (h:min). White arrowheads indicate successful mitosis and cell division; Red arrowheads indicate stable proliferation of daughter cells over three generations; Yellow arrowheads indicate failed mitotic entry; Black arrowheads indicate cell death. Bars, 50 μm.

To track the fate of CNF1-induced polyploid cell, we labeled cells with the membrane permeable DNA dye Ruby stain without affecting cell viability and sorted polyploid cells (DNA content >4 C) using FACS (Fig. 2a). The sorted polyploid cells were cultured in fresh medium without CNF1 for two weeks. After 3 days recovery, we found that some polyploid cells started to re-enter a normal cell cycle. As showed in Fig. 2b and Supplementary Movie S3, a binucleated cell showed successful mitotic re-entry with mitotic rounding (06:10, white arrowhead), followed by cytokinesis to form two daughter cells (06:40 and 07:10, white arrowheads). Meanwhile, a mononucleated polyploid cell showed mitotic rounding (06:10 white arrowhead) and subsequent tripolar cell division (06:40 and 07:10 white arrowheads). Then two of the daughter cells fused together and formed a multinucleated cell (09:10 white arrowhead). Although the first-generation progeny of CNF1-induced polyploid cells were still a mixture of mononucleated and multinucleated cells, and exhibited a giant and flattened cell shape, the daughter cells acquired the ability to override the arrest of proliferation and underwent further cell division (Supplementary Movie S3). In contrast to successful mitotic re-entry, some polyploid cells failed to re-enter mitosis and proceeded to apoptosis-like cell death (08:50 to 20:20, black arrowheads).

After one-week recovery, the population of polyploid cell-derived daughter cells increased steadily and became to dominate in the total cell population. As shown in Fig. 2c and Supplementary Movie S4, the daughter cells from different areas underwent normal mitosis and efficient proliferation (white arrowheads). Representative cell division through three cell generations was observed and marked with red arrowheads. Several asymmetric/tripolar cell division (10:20, white arrowheads) and cell death in generated daughter cells (23:40, black arrowheads) were observed. In contrast to proliferative daughter cells, two co-existed polyploid cells showed morphology of mitotic rounding or even cytokinesis but failed to separate into daughter cells (00:00 to 06:10, yellow arrowheads). One polyploid giant cell showed eventually necrosis-like cell death (48:40, black arrowhead).

After two weeks recovery, no giant cell was found in the culture under light microscopy. We collected the cells and performed DNA content analysis. In line with the microscopic observations, the polyploid population (DNA cotent >4 C) of HCT-116 decreased to the level of untreated cells (Supplementary Fig. S2). These findings suggest a survival strategy of these cells through polyploidisation and subsequent depolyploidisation.

### Multinucleated senescent arrest and escape

**Figure 3:**
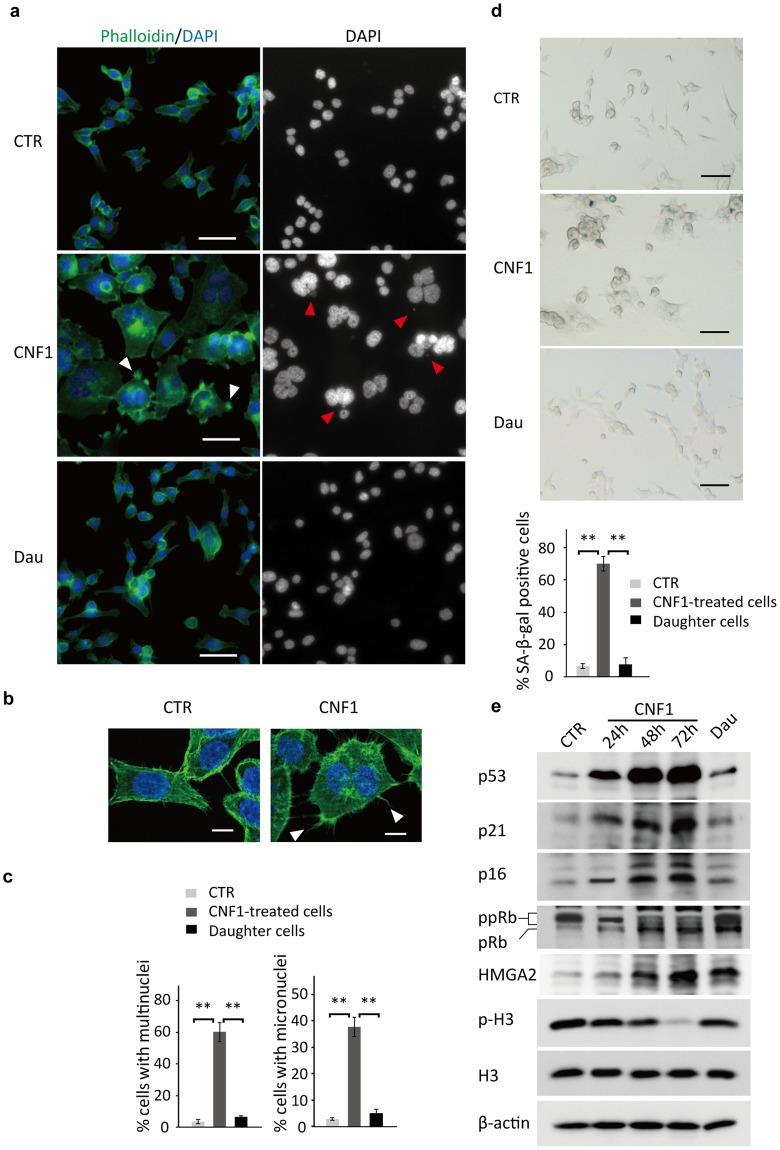
Characterisation of multinucleated senescent arrest and escape. (**a**) Representative fluorescence images of HCT-116 cells untreated (CTR) or treated with 5 nM CNF1 for 72 h (CNF1), or polyploid HCT-116 cell-derived daughter cells (Dau), stained with phalloidin and DAPI. White arrowheads indicate filopodium-like structure at the cell periphery; Red arrowheads indicate micronuclei in the multinucleated cells. Bars, 50 μm. (**b**) Representative confocal images of HCT-116 cells untreated (CTR) or treated with 5 nM CNF1 for 72 h (CNF1). White arrowheads indicate filopodium-like structure at the cell periphery. Bars, 10 μm. (**c**) Percentage of cells containing multinuclei or micronuclei in HCT-116 cells untreated or treated with 5 nM CNF1 for 72 h, or polyploid HCT-116 cell-derived daughter cells. Data are mean ± SD of three independent experiments. **p < 0.01. (**d**) Representative images of SA-β-gal staining of HCT-116 cells (upper panel) and percentage of SA-β-gal positive cells (lower panel). Bars, 50 μm. Data are mean ± SD of three independent experiments. **p < 0.01. (e) Representative immunoblot analysis of p53, p21, p16, pRb, HMGA2, p-H3, H3 and β-actin in HCT-116 cells untreated or treated with 5 nM CNF1 at 24 h, 48 h and 72 h, or polyploid HCT-116 cell-derived daughter cells.

To further understand the process of polyploidisation and depolyploidisation upon CNF1 treatment, cells were stained with phalloidin and DAPI to visualise cell cytoskeleton and nuclei, respectively, using fluorescent microscopy. In line with the observation of time-lapse phase contrast microscopy, HCT-116 cells showed enlarged, abnormal cell shape with peripheral filopodia (Fig. 3a, white arrowheads) in the merged image with F-actin cytoskeleton (green) and nuclei (blue). We further confirmed that CNF1-treated HCT-116 cells formed more filopodia than untreated cells using confocal microscopy (Fig. 3b, white arrowheads), suggesting the activation of Rho family GTPase Cdc42 by CNF1 as described in the earlier studies^9^. DAPI staining showed that some nuclei were clustered together in CNF1-treated HCT-116 (Fig. 3a) and 60% of cells exhibited multinucleation after 72 h exposure to CNF1 (Fig. 3c). By contrast, cells carrying two or more nuclei were less than 4% among untreated cells and 7% among polyploid cell-derived daughter cells (Fig. 3a,c). These findings confirm the cells undergo multinucleation-associated polyploidisation upon CNF1 treatment and survive through depolyploidisation. Moreover, the DAPI stain also revealed the presence of micronuclei in multinucleated cells. Cell micronucleus is formed as a result of chromosome damage and served as a biomarker of genomic instability^39^. The frequency of micronuclei formation (Fig. 3a, red arrowheads) increased significantly after CNF1 treatment. Approximately 38% of CNF1-treated HCT-116 carried one or more micronuclei, whereas less than 8% of occurrence was observed in untreated cells and polyploid cell-derived daughter cells (Fig. 3c), implying that nuclear abnormality upon CNF1 treatment might cause genomic instability in these cells.

Due to the inhibitory role of CNF1 in cell proliferation, we suggest that CNF1-induced polyploid cells might undergo proliferative arrest. A recent study showed that mouse glioma GL261 cells after CNF1 treatment were positive for senescence-associated β-galactosidase (SA-β-gal) activity, a biomarker of senescence, suggesting that senescent arrest might be the consequence of CNF1-induced polyploidisation^40^. To test this hypothesis, we checked the activity of SA-β-gal in HCT-116. As shown in Fig. 3d, 70% of HCT-116 cells showed increased SA-β-gal activity after exposure to CNF1 for 72 h, whereas less than 9% of untreated and polyploid cell-derived daughter cells were positive for SA-β-gal activity.

To confirm CNF1-induced senescent arrest, we analysed the expression of cell-cycle checkpoints proteins and observed that the expression of p53, p21 and p16 in HCT-116 cells were significantly increased after 24 h exposure to CNF1, and further increased after prolonged exposure during 72 h as compared with untreated control cells (Fig. 3e). We further determined the expression of the retinoblastoma tumor suppressor protein (pRb), a central senescence regulator acting downstream of p53, p21 and p16 pathways^41^. The level of hyperphosphorylated pRb (ppRb) clearly decreased in HCT-116 at 24 h after exposure to CNF1, conversely the level of hypophosphorylated or unphosphorylated pRb increased significantly after prolonged exposure of 48 h and 72 h, suggesting that CNF1 causes senescent arrest through p53/p21 and p16 mediated inhibition of pRb phosphorylation. We also found that the expression of high mobility group A2 protein (HMGA2) increased after 48 h and 72 h exposure to CNF1, implying HMGA2-induced formation of senescence associated heterochromatin foci (SAHF) during senescence (Fig. 3e). Accordingly, the expression of the mitotic marker phospho-histone H3 ser10 (P-H3) decreased tremendously in CNF1-treated cells at 48 h and 72 h in comparison with untreated control cells.

Our data also revealed the process of depolyploidisation-associated cell survival and it suggests that CNF1-induced senescence in the HCT-116 cells is a transient cell cycle arrest that may be overridden by re-entering into a normal cell cycle. Indeed, polyploid cell-derived daughter cells did not show striking changes in either cell-cycle checkpoints proteins or P-H3, which is consistent with microscopic observations of cell proliferation. However, the levels of hypophosphorylated or unphosphorylated pRb and HMGA2 still remained higher in daughter cells than those of untreated control cells.

Taken together, our data suggest that CNF1 can cause cell cycle blockade and mitotic exit, eventually resulting in senescent arrest. However, polyploid cells only showed transient senescent arrest, and recovered rapidly from CNF1 damage through depolyploidisation and restore normal ability of proliferation.

### CNF1 causes genomic instability in polyploid cell-derived daughter cells

**Figure 4:**
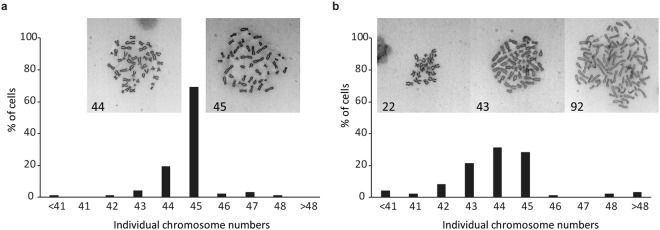
CNF1 induces genomic instability. Metaphase chromosome spread analysis of untreated HCT-116 cells (**a**) and polyploid HCT-116 cell-derived daughter cells (**b**). n = 100 each group. Representative images of chromosome spread with counted chromosome number were shown.

We showed that cells formed micronuclei during polyploidisation upon CNF1 treatment, and multinucleated polyploid cells underwent asymmetric cell division during depolyploidisation, both of which are markers of genomic instability. To assess if CNF1 treatment affected the genomic stability and led to aneuploid progeny, we performed chromosome spread analysis of daughter cells. As shown in Fig. 4a, untreated HCT-116 cells exhibited a near-diploid pattern with 69% of metaphases of cells carrying 45 or 44 chromosomes, which is in accordance with cytogenetic analysis of HCT-116 in earlier studies^42,43^. However, the polyploid cell-derived daughter cells showed striking difference in the distribution of cells with near-diploid chromosomes. The rate of cells carrying 45 chromosomes decreased to 28% and those with 44 chromosomes increased to 31% (Fig. 4b). Moreover, 41% of daughter cells had more than 45 or less than 44 chromosomes in comparison with only 12% observed in untreated HCT-116 cells. Taken together, these results suggest that CNF1 treatment increases the incidence of chromosome aneuploidy and enhances genomic instability in depolyploidised daughter cells.

### Polyploid cell-derived daughter cell exhibits enhanced resistance to CNF1

**Figure 5:**
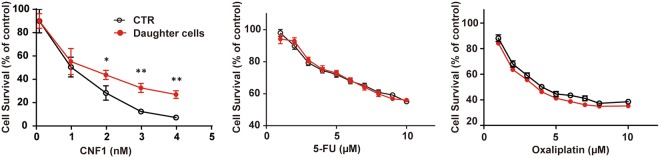
Polyploid HCT-116 cell-derived daughter cells show enhanced resistance to CNF1, but no significant alteration of chemoresistance. Clonogenic assay of HCT-116 cells and polyploid HCT-116 cell-derived daughter cells after CNF1 treatment; MTS assay of HCT-116 cells and polyploid HCT-116 cell-derived daughter cells after 5-FU or oxaliplatin treatment. Data are mean ± SD of three independent experiments. *p < 0.05; **p < 0.01.

Interestingly, in our study, we observed that CNF1-induced polyploid cells exhibit the phenotype reminiscent of therapy-induced cellular senescence, which is believed to be a mechanism to favor cancer cell escape from chemotherapy-induced death and simultaneously confer resistance to anticancer therapy^41,44^. We then performed proliferation assay to determine whether polyploid cell-derived daughter cells acquire resistance to CNF1 re-treatment or not. In comparison to untreated control cells, the HCT-116 daughter cells significantly increased the resistance to CNF1, especially at higher concentrations (Fig. 5). Surprisingly, the HCT-116 daughter cells did not show any significant increase in resistance to 5-fluorouracil and oxaliplatin, the two commonly used drugs in chemotherapy against colon cancer^45^. The antiproliferative activity of 5-fluorouracil and oxaliplatin remained equal in daughter cells and untreated control cells, suggesting that CNF1 does not promote resistance to agents/drugs used in chemotherapy.

## Discussion

In this study, we revealed the biological process of multinucleated polyploidisation in human colonic epithelial cells after treatment with a lethal dose of CNF1. Moreover, for the first time, we observed the survival of CNF1-treated cells through depolyploidisation and the formation of genomically unstable progeny.

Multinucleation and cell shape enlargement are classic morphologic changes that are reported in various cell lines after CNF1 treatment^10^. In this study, the time-lapse imaging analysis suggests that multinucleation and cell shape enlargement are the consequence of cell endoreplication. As a variant of normal mitotic cell cycle, endoreplication is an evolutionarily conserved manner to generate multinucleated polyploidy in many different organisms including plants, animals and insects etc^46^. It can be part of either developmental programme or physiological stress response. In this case, we suggest that HCT-116 cells switch to endoreplication as a compromised response when encountering CNF1-induced mitotic stress and cytokinesis failure. Both the increasing proportion of polyploid cells (DNA content >4 C) and the immunoblotting detection of mitosis marker, phosphorylated histone H3 suggest an active endomitosis during early stage of endoreplication.

Several reports suggest that endoreplication and polyploidisation will drive cells to senescent arrest^43,47,48^. It was demonstrated that mouse glioma GL261 cells, after CNF1 treatment, displayed positive SA-β-gal activity, a senescence biomarker^40^. We observed that CNF1-treated HCT-116 cells have an increased SA-β-gal activity. Moreover, expression of p53, p21 and p16, markers that are controlling senescence, was strongly induced after CNF1 treatment, in spite of HCT-116 possessing a silent p16 gene^49^. Consistently, pRb phosphorylation was suppressed by CNF1 treatment. Furthermore, the ceased expression of the mitosis marker phosphorylated histone H3 confirmed that cells in senescent arrest exited endomitosis.

Cellular senescence, like apoptosis, has been proposed to be a tumor-suppressor mechanism that irreversibly arrests cell growth^41^. However, recent studies point out that polyploid senescent cells can escape the senescent arrest and re-enter into a cell cycle^50–52^. In this study, we demonstrated that CNF1-induced senescence is also a reversible growth arrest. In prolonged culture with fresh medium, a subset of polyploid cells re-entered into a cell cycle, depolyploidised often through asymmetric cell division, and eventually generated more aneuploid progeny. Aneuploidy is the most common feature of human solid tumors. Increased aneuploidy or genomic instability might contribute to tumorigenesis^53^. However, there is still a big gap to be filled in our understanding, whether CNF1 contributes to colon tumorigenesis or not, requiring further characterisation of the aneuploid daughter cells.

CNF1-induced polyploidisation and depolyploidisation might be considered a reminiscent of therapy-induced cellular senescence and escape, which are linked to clinical chemoresistance and cancer relapse^54,55^. Similarly, we found that depolyploidised daughter cells showed a significant increase in resistance to CNF1. However, our data do not support that daughter cells acquire resistance to DNA-damaging drugs, suggesting that the resistance mechanism towards CNF1 might be different from the resistance mechanisms to DNA-damaging drugs. Despite that the daughter cells have obtained capacity of proliferation, it seems that the daughter cells inherit characteristics from senescent parental cells, such as higher expression levels of hypophosphorylated or unphosphorylated pRb and HMGA2. Whether the alterations of pRb and HMGA2 expression result in an increase of CNF1 resistance requires further examination. It is worth noting that CNF1 processes a promising potential in therapy for glioma tumor, neuropathic pain and other neurological disorders^56^, whether the therapeutic treatment will confer resistance to CNF1 in host cell or tissue remains unknown. Further deciphering the resistance mechanism to CNF1 will not only help to understand the role of CNF1 in cell fate determination, but may also enable the identification of novel therapeutic agents indifferent to these resistance mechanisms.

In addition to CNF1, bacteria also produce other CNF-like proteins, including CNF2 and CNF3 from *E. coli* strains isolated from animals^57^, CNFY from *Yersinia pseudotuberculosis*^19^ and Bordetella dermonecrotic toxin (DNT)^21^. CNF homologous gene is also present in the genome of pathogenic marine bacteria including *Moritella viscosa*^58^ and *Photobacterium damselae*^59^. Although CNFs share an identical mode of action in modification of host small GTPases and have a similar activity to DNT, CNF-like proteins exhibit distinct specificity and preference for the target small GTPases^2,57^, which might lead to a different cell fate modulation by CNF-like proteins. It was shown that CNFY induces cleavage of apoptosis marker PARP and increases caspase-3/7 activity in LNCaP cells, while CNF1 does not^60^. Whether CNF-like proteins have a similar effect to CNF1 in cell fate modulation requires further investigation.

In summary, we suggest a pathway mechanism implying that CNF1 is reprogramming host cell fate toward survival through multinucleation/polyploidisation, reversible senescent arrest and subsequent depolyploidisation. The increased genomic instability in surviving descendent cells may have the implication to playing a potential role of CNF1 in carcinogenesis.

## Methods

### Cell Culture and CNF1 Treatment

The HCT-116 human colon cancer cell line was kindly provided by Dr. Bert Vogelstein (Johns Hopkins University, Baltimore, MD, USA). Cells were cultured in Dulbecco’s modified Eagle’s medium (DMEM; Gibco, Thermo Fisher Scientific, Waltham, MA, USA) supplemented with 10% fetal bovine serum (Sigma-Aldrich, St. Louis, MO, USA). CNF1 was recombinantly expressed from *E. coli* M15/pREP4 transformed with pCNF24 plasmids that were kindly provided by Dr. Alison D. O’Brien (Uniformed Services University, Bethesda, MD, USA). CNF1 purification was performed as described previously^61^. HCT-116 cells were seeded at a density of 5 000/cm^2^ 24 h before CNF1 treatment. Cells were cultured in medium with the indicated concentrations of CNF1 for 72 h, and then allowed to recover for 14 days in fresh medium without CNF1.

### Clonogenic Assay

To assess the effect of CNF1 on cell proliferation, a clonogenic assay was performed as previously described with minor changes^40,62^. Briefly, HCT-116 cells were seeded onto 24-well plates with a density of 500 cells/well overnight, and then incubated with increasing concentrations of CNF1 (0.1–4 nM) for 10 days. The number of colonies (>50 cells) was assessed in triplicate wells by crystal violet staining. IC50 was determined by nonlinear regression using Sigmaplot (Systat Software Inc., San Jose, CA, USA).

### FACS Analysis and Cell Sorting

For fluorescence activated cell sorting (FACS)-based DNA content analysis, Cells were dissociated by TrypLE Select (Gibco, Thermo Fisher Scientific) and fixed with 70% ethanol. Samples were washed with PBS and resuspended in PBS containing 50 μg/ml propidium iodide and 100 μg/ml RNase A, and then incubated for 30 min at 37 °C. DNA content was determined by a LSR II flow cytometry and analysed using FACSDiva software (BD Biosciences, San Jose, CA, USA).

For FACS cell sorting, live cells were incubated with Ruby stain (Thermo Fisher Scientific) at 37 °C for 15 min, and then analysed on a FACSAria III cell sorter (BD Biosciences). Polyploid cells (DNA content >4 C) were sorted and collected according to the intensity of Ruby stain. Sorted cells were cultured in fresh DMEM medium in preparation for live-cell imaging.

### Microscopy and Image Processing

Long-term time-lapse imaging was performed on an Eclipse Ti-E inverted microscope (Nikon Instruments, Amsterdam, The Netherlands) using a temperature- and humidity-controlled chamber (Okolabs, Pozzuoli, Italy) with 5% CO2 at 37 °C. HCT-116 cells were seeded on 35-mm dishes with low density and phase contrast images were captured every 10 minutes. Images and videos were analysed by NIS elements AR software (Nikon Instruments).

Fluorescence and confocal microscopy were performed as previously described^63,64^. Briefly, cells were grown on cover slip and fixed with 2% paraformaldehyde (Sigma-Aldrich). Actin cytoskeletons and nuclei were stained with Alexa Fluor 488 phalloidin (Thermo Fisher Scientific) and DAPI (Sigma-Aldrich), respectively. Fluorescence microscopy was performed on an Eclipse 90i microscope (Nikon Instruments) equipped with a 12-bit, black and white CCD camera (Hamamatsu Photonics, Hamamatsu City, Japan). Images were analysed by NIS elements AR software (Nikon Instruments). Cell counting was performed in at least five fields of triplicate cover slips. Confocal microscopy was performed on a D-Eclipse C1 confocal microscope (Nikon Instruments). Images were processed by EZ-C1 Freeviewer software (Nikon Instruments).

### SA-β-Gal Staining

Senescence-associated β-galactosidase (SA-β-gal) staining was performed using a senescence cells histochemical staining kit (Sigma-Aldrich) according to the manufacturer’s instruction. Cells were evaluated under a light microscope and the SA-β-gal positive cells were counted in at least five fields of triplicate plates.

### SDS-PAGE and Immunoblot Analysis

Whole cell extracts were prepared in RIPA cell lysis buffer (10 mM Tris-HCl pH 8.0, 140 mM NaCl, 1 mM EDTA, 0.5 mM EGTA, 1% Triton X-100, 0.1% SDS, 0.1% Na-deoxycholate) containing protease and phosphatase inhibitors (Roche Life Science, Penzberg, Germany). The samples were separated by sodium dodecyl sulfate-13% polyacrylamide gel electrophoresis (SDS-PAGE), and then blotted onto a PVDF membrane (Amersham, GE Healthcare Life Sciences, Little Chalfont, UK). Immunoblot was performed as previously described^65^ using antibodies from following sources: anti-p53 (DO-1; Santa Cruz Biotechnology, Dallas, TX, USA), anti-p21 (F-5; Santa Cruz Biotechnology), anti-p16 (BD Biosciences), anti-human retinoblastoma protein (pRb; BD Biosciences), anti-phospho-histone H3 Ser10 (p-H3, Cell Signaling Technology, Danvers, MA, USA), anti-histone H3 (Merck Millipore, Darmstadt, Germany) and anti-β-actin (Sigma-Aldrich). Horseradish peroxidase (HRP)-conjugated donkey anti-rabbit immunoglobulin (Ig) G (AgriSera AB, Vännäs, Sweden) or goat anti-mouse IgG (Dako, Glostrup, Denmark) was used as a secondary antibody. Immunoreactive bands were visualized using a Clarity Western ECL substrate kit (Bio-Rad, Hercules, CA, USA) on a LAS-3000 luminescent image analyser (GE Healthcare).

### Metaphase Chromosome Spread Analysis

To arrest cells at metaphase, cells were treated with 0.1 μg/ml colcemid (Thermo Fisher Scientific) for 4 h. After that, cells were collected by mitotic shake off and treated with hypotonic solution (75 mM KCl) for 15 min at 37 °C. Cells were fixed in an ice-cold mixture of methanol and acetic acid (3:1) for three times. Cells were then dropped on glass slides, air-dried and immediately stained with a 3% Giemsa solution (Sigma-Aldrich) for 1 min, and then rinsed with water. Glass slides were examined under a light microscope and 100 metaphase spreads were counted.

### MTS Assay

Cell viability in response to 5-fluorouracil or oxaliplatin was determined using 3-(4,5-dimethylthiazol-2-yl)-5-(3-carboxymethoxyphenyl)-2-(4-sulfophenyl)-2H-tetrazolium (MTS) in the CellTiter 96 aqueous one solution cell proliferation kit (Promega, Madison, WI, USA) according to the manufacturer’s instruction. Cells were seeded on 96-well plates (5 000 cells/well) and treated with different concentrations of 5-fluorouracil (5-FU) or oxaliplatin for 72 h. The absorbance at 490 nm was measured on an Infinite M200 microplate reader (Tecan, Mannendorf, Switzerland).

### Statistical analysis

Data are indicated as means ± standard deviation (SD) and are representative of three independent experiments. Statistical comparisons were made with a two-tailed Student’s *t* test. *p* < 0.05 was considered to be significant.

## Electronic supplementary material

Supplementary Information

Supplementary Video 1

Supplementary Video 2

Supplementary Video 3

Supplementary Video 4

## Data Availability

All data are available from the corresponding authors upon reasonable request.
