# Supplementary material for: Reversible senescence of human colon cancer cells after blockage of mitosis/cytokinesis caused by the CNF1 cyclomodulin from Escherichia coli
Source: Sci Rep. 2018 Dec 12;8:17780. doi: 10.1038/s41598-018-36036-5 (PMC6290797; doi:10.1038/s41598-018-36036-5)
Supplement: Supplementary file 1 — Supplementary Information [file 41598_2018_36036_MOESM1_ESM.pdf]

Supplementary Information

# Reversible senescence of human colon cancer cells after blockage of mitosis/cytokinesis caused by the CNF1 cyclomodulin from *Escherichia coli*

**Zhen Zhang[1#], Kyaw Min Aung[1], Bernt Eric Uhlin[1], Sun Nyunt Wai[1#]**

**[1]Department of Molecular Biology and The Laboratory for Molecular Infection Medicine Sweden (MIMS) Umeå University, SE-90187 Umeå, Sweden.**

**# Corresponding authors:**

**E-mail: sun.nyunt.wai@umu.se**

**E-mail: zhen.zhang@helsinki.fi**

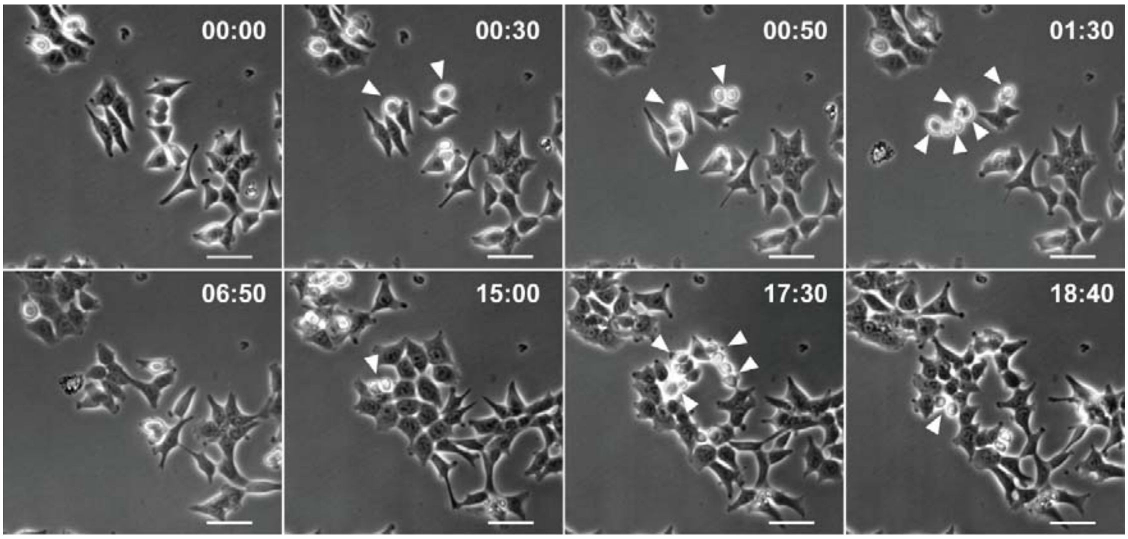


**Figure S1.** Representative time-lapse images of proliferation in HCT-116 without CNF1 treatment. Time is indicated in hours and minutes (h:min). White arrowheads indicate cells undergoing mitosis and cell division. Bars, 50 μm.

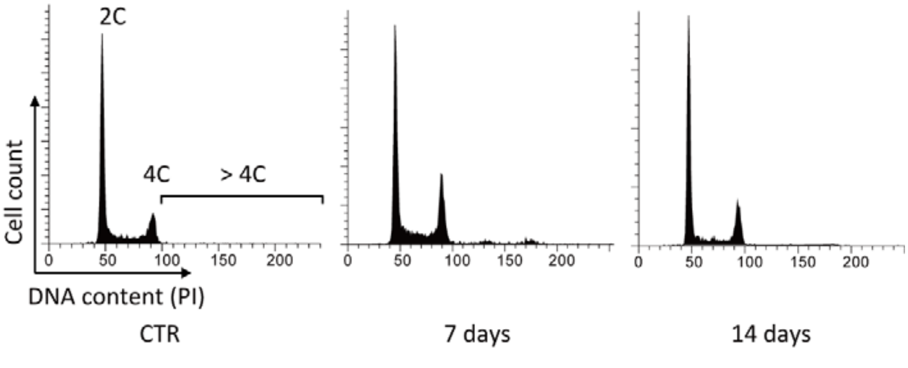


**Figure S2.** DNA content analysis of HCT-116 untreated cells and polyploid HCT-116 cell-derived daughter cells after 7 and 14 days recovery.

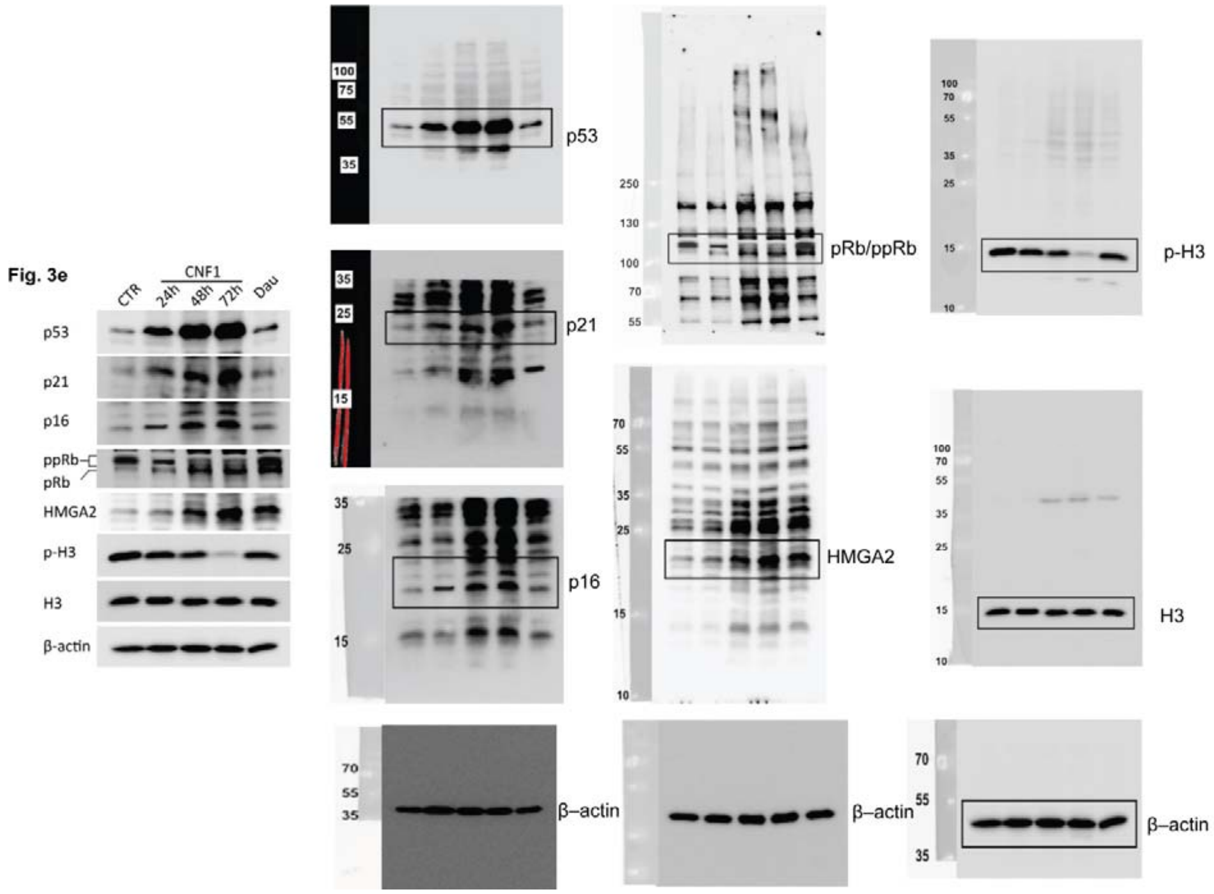


**Figure S3.** Full-length immunoblots of antibodies that are tested in this study. The immunoreactive bands are determined with protein marker. The cropped area of immunoblot in Fig. 3e is indicated by a black rectangle. The β–actin antibody has been three times, did not show any significant difference. One representative immunoblot of β–actin is presented in Fig. 3e.

**Supplementary Movie Legends**

**Movie S1.** Representative time-lapse movie of CNF1-induced endoreplication in HCT-116. Time is indicated in hours and minutes (h:min). Bars, 50 μm.

**Movie S2.** Representative time-lapse movie of proliferation in HCT-116 without CNF1 treatment. Time is indicated in hours and minutes (h:min). Bars, 50 μm.

**Movie S3.** Representative time-lapse movie of depolyploidisation of polyploid HCT-116 cells in the first 7 days after CNF1 treatment. Time is indicated in hours and minutes (h:min). Bars, 50 μm.

**Movie S4.** Representative time-lapse images of depolyploidisation of polyploid HCT-116 cells in the second 7 days after CNF1 treatment. Time is indicated in hours and minutes (h:min). Bars, 50 μm.
